# From Mouse to Human: Cellular Morphometric Subtype Learned From Mouse Mammary Tumors Provides Prognostic Value in Human Breast Cancer

**DOI:** 10.3389/fonc.2021.819565

**Published:** 2022-02-11

**Authors:** Hang Chang, Xu Yang, Jade Moore, Xiao-Ping Liu, Kuang-Yu Jen, Antoine M. Snijders, Lin Ma, William Chou, Roberto Corchado-Cobos, Natalia García-Sancha, Marina Mendiburu-Eliçabe, Jesus Pérez-Losada, Mary Helen Barcellos-Hoff, Jian-Hua Mao

**Affiliations:** ^1^ Biological Systems and Engineering Division, Lawrence Berkeley National Laboratory, Berkeley, CA, United States; ^2^ Berkeley Biomedical Data Science Center, Lawrence Berkeley National Laboratory, Berkeley, CA, United States; ^3^ Key Laboratory of Modern Toxicology of Ministry of Education, School of Public Health, Nanjing Medical University, Nanjing, China; ^4^ Department of Radiation Oncology and Helen Diller Family Comprehensive Cancer Center, University of California, San Francisco, San Francisco, CA, United States; ^5^ Department of Pathology and Laboratory Medicine, School of Medicine, University of California, Davis, Davis, CA, United States; ^6^ Instituto de Biología Molecular y Celular del Cáncer, Universidad de Salamanca/Consejo Superior de Investigaciones Científicas (CSIC), Salamanca, Spain; ^7^ Instituto de Investigación Biosanitaria de Salamanca, Salamanca, Spain

**Keywords:** mouse mammary tumor, metastasis, human breast cancers, transfer learning, cellular morphometric biomarkers, cellular morphometric subtypes, overall survival (OS)

## Abstract

Mouse models of cancer provide a powerful tool for investigating all aspects of cancer biology. In this study, we used our recently developed machine learning approach to identify the cellular morphometric biomarkers (CMB) from digital images of hematoxylin and eosin (H&E) micrographs of orthotopic *Trp53*-null mammary tumors (n = 154) and to discover the corresponding cellular morphometric subtypes (CMS). Of the two CMS identified, CMS-2 was significantly associated with shorter survival (p = 0.0084). We then evaluated the learned CMB and corresponding CMS model in *MMTV-Erbb2* transgenic mouse mammary tumors (n = 53) in which CMS-2 was significantly correlated with the presence of metastasis (p = 0.004). We next evaluated the mouse CMB and CMS model on The Cancer Genome Atlas breast cancer (TCGA-BRCA) cohort (n = 1017). Kaplan–Meier analysis showed significantly shorter overall survival (OS) of CMS-2 patients compared to CMS-1 patients (p = 0.024) and added significant prognostic value in multi-variable analysis of clinical and molecular factors, namely, age, pathological stage, and PAM50 molecular subtype. Thus, application of CMS to digital images of routine workflow H&E preparations can provide unbiased biological stratification to inform patient care.

## Introduction

Breast cancer (BC) is the most commonly diagnosed cancer in women, and is the second leading cause of cancer death among women in the U.S. Breast cancer is highly heterogeneous in regard to molecular events, histological properties, and clinical outcomes; hence, stratification of BC is mandatory for precision diagnosis, prognosis, and treatment of patients. Currently, the presence of estrogen receptor (ER), progesterone receptor (PR), and human epidermal growth factor receptor 2 (HER2) are used to make many clinical decisions about treatment. The molecular landscape has also been used to classify BC into genomic subtypes that have prognostic value (1, 2). For example, one of the first classifications based on microarray analysis of gene expression describes five subtypes, namely, luminal A, luminal B, HER2 positive, basal-like, and normal-like, and showed significantly different outcomes across subtypes (3). Other genomic tests report the activity of specific genes to predict the likelihood of recurrence or metastatic progression to aid clinical decision-making. The prognostic implication of genetic alterations, such as *BRCA1*, *BRCA2*, and *PALB-2* germline mutations, are also widely used in women with familial history of breast cancer (4). But the oldest and most basic classification is that by microscopic examination of the histology that encompasses the morphological features of cancer cells, their distribution, and the organization of local surrounding tissue. The World Health Organization classification recognizes at least seventeen distinct histological subtypes; thus, the complexity and variety of the disease warrants an automated, unbiased approach.

Mouse models have an essential role in cancer research to understand the genetic basis of mammary tumor development and progression, to investigate consequences of frequent mutations, to study the processes leading to and impacting tumor development, and to identify and test putative therapies (5–7). As in BC, mouse mammary cancer is diverse in terms of histological characteristics and molecular and genetic alterations. Human breast cancer diversity is particularly well modeled in carcinomas arising in transgenic mice lacking expression of *Trp53* in mammary epithelium (8).

We recently developed an artificial intelligence (AI) framework (CMS-ML: Cellular morphometric subtyping *via* machine learning) to identify cellular morphometric biomarker (CMB) used to discover cellular morphometric subtype (CMS). We applied this framework to low grade gliomas, and discovered CMS associated with specific molecular alterations, immune microenvironment and prognosis (9). In the present study, we used the same framework to identify CMB and discover CMS from digital images of standard hematoxylin and eosin (H&E) staining of mouse mammary tumors. We then evaluated the CMB and CMS learned from one mouse mammary tumor model to interrogate a different mouse mammary tumor model and then applied these to human BC. Our analyses showed that the application of CMS-ML to digital images of routine workflow H&E preparations provide unbiased biological stratification that can be used to inform patient care.

## Methods

### Animal Models

Two animal models were used in this study, i.e., orthotopic*Trp53*-null mammary transplant model (referred to herein as *Trp53*-null) and *MMTV-Erbb2* transgenic mouse model (referred to herein as *Erbb2*). In *Trp53*-null model, mammary tumors were developed from transplants of non-irradiated *Trp53* ^−/−^ BALB/c mammary gland fragments into cleared fat pads of female F1Bx hosts, where F1Bx mice were the progeny of the cross (BALB/c × SPRET/EiJ) F1 × BALB/c (10). The work related to *Trp53*-null model was approved by the Animal Welfare and Research Committee at Lawrence Berkeley National Laboratory. For the *Erbb2* model, mammary tumors were derived from a female F1BX population of the progeny of the cross (C57BL/6 × FVB/N *MMTV-Erbb2*) F1 × FVB/N. Mice with only one primary mammary tumor were selected to ensure that the metastatic foci were from that tumor (11). The work related to *Erbb2 model* was approved by the Institutional Animal Care and Bioethical Committee of Universidad de Salamanca. We leveraged data derived from two previous studies (10, 11), and did not carry out any additional animal work.

### Data Collection

The training data was digital images of H&E-stained sections of formalin-fixed paraffin-embedded (FFPE) *Trp53*-null mammary tumors (**Supplementary Table 1**) (10). Tumors were detected by palpation. Upon detection, tumor size was measured using digital calipers. For tumor bearing mice, survival was defined as the time from tumor onset (tumor size about 2 × 2 mm) to euthanasia (tumor size reaches 10 × 10 mm). The independent validation specimens and associated data were mammary tumors from the *MMTV-Erbb2* transgenic model (**Supplementary Table 2**) (11). Distant metastases were detected by gross evaluation and histopathology. Only mice with a single primary mammary tumor were selected for this study to ensure that the metastatic foci originated from this tumor. The patient data consisted of digital micrographs of diagnostic H&E histology slides and the corresponding clinical information from The Cancer Genome Atlas Breast Invasive Carcinoma (TCGA-BRCA) cohort (**Supplementary Tables 3**, **4**). The inclusion criteria were primary BRCA with diagnostic slides and overall survival (OS) information available. Note, diagnostic slides without magnification information were excluded.

### Identification of Cellular Morphometric Biomarkers

We developed AI pipeline based on stacked predictive sparse decomposition (SPSD) (12) to discovery the underlying cellular morphometric characteristics from the 15 cellular morphometric features extracted from the diagnostic micrographs of H&E stained sections of FFPE mouse tumors (one slide per mouse per tumor), and thereafter identified 256 Cellular Morphometric Biomarkers (CMB) for cellular object representation. Specifically, in this study, we used a single network layer with 256 dictionary elements (i.e., CMB) and sparsity constraint 30 at a fixed random sampling rate of 1,000 cellular objects per whole slide image of tumor histology from the mouse training cohort. The pre-trained SPSD model reconstructs each cellular region (represented as a vector of 15 morphometric properties) as a sparse combination of pre-identified 256 CMB, and after that represents each cellular object as the sparse code (i.e., sparse coefficients) during reconstruction, where the sparsity constraint leads to the reconstruction mainly contributed by the top 30 CMB.

### Clinical and Biological Evaluation of Cellular Morphometric Biomarkers

We evaluated the prognostic impact of representative CMB with the most prominent variations mined from mouse cohort with Cox proportional hazards regression (CoxPH) model (survival package in R, Version 3.2-3). Also, we examined the effects of high or low levels of representative prognostic significant CMB on OS using Kaplan–Meier analysis (survminer package in R, Version 0.4.8) and log-rank test (survival package in R, Version 3.2-3), where both mouse cohorts and the TCGA-BRCA cohort were divided into CMB-high and CMB-low groups per CMB (survminer package in R, Version 0.4.8).

### Construction of Mouse-/Patient-Level Cellular Morphometric Context Representation

The mouse-/patient-level cellular morphometric context representation was constructed based on pre-identified 256 CMB as an aggregation (i.e., max-pooling) of all the cellular sparse codes extracted *via* pre-built SPSD model from the cellular objects belonging to the digital micrographs of H&E stained, FFPE diagnostic slides of the same patients. Specifically, it consists of steps as follows, (1) delineation of cellular architecture and extraction of cellular morphometric properties from diagnostic slides of each mouse/patient; (2) construction of cellular sparse codes for the cellular objects belonging to each mouse/patient based on pre-identified 256 CMB and pre-built SPSD model; and (3) aggregation (i.e., max-pooling) of all cellular sparse codes belonging to the same patient to form the mouse-/patient-level cellular morphometric representation.

### Identification of Mouse CMS and Translation to Human Breast Cancer

The mouse mammary tumor subtype was identified based on mouse-level cellular morphometric context representation through consensus clustering strategy (13) (ConsensusClusterPlus package in R, Version 1.50.0) with k-mean clustering, Euclidean distance, and 500 bootstrapping iterations; and the optimal number of subtypes was determined by the consistency of cluster assignment (consensus matrix) and the prognostic impact of subtypes. During mouse subtype translation, for a human breast cancer patient, the subtype was assigned as follows: (1) construct patient-level cellular morphometric context representation with pre-built CMB and SPSD model; (2) calculate the Euclidean distances between the representation of the new patient and the mean representation of each pre-identified mouse mammary tumor subtype; and (3) assign the new patient to its closest subtype yielding smallest Euclidean distance. During fine-tuning, the human breast cancer subtype was identified based on patient-level cellular morphometric context representation (built upon CMB learned from mouse mammary tumors) through consensus clustering strategy (13) (ConsensusClusterPlus package in R, Version 1.50.0).

### Clinical Evaluation of Patient Subtype

The latest clinical data of the TCGA-BRCA cohort was downloaded from Genomic Data Commons (GDC, https://portal.gdc.cancer.gov/), and the CMS translation from mouse to each breast cancer patient in the TCGA-BRCA cohort was achieved through the application/refinement of pre-built mouse mammary tumor subtype model as described previously. The translational relevance was assessed as follows: (1) the prognostic impact of patient CMS subtype was evaluated on the OS of the TCGA-BRCA cohort with univariate and stepwise multivariate Cox proportional hazards regression (CoxPH) models (survival package in R, Version 3.2-3), and the subtype-specific survival was visualized through Kaplan–Meier curve (survminer package in R, Version 0.4.8); (2) survival prediction, based on multivariate CoxPH model, was evaluated for the prediction of 5-, 10-, and 20-year survival rates of BRCA patents, where the multivariate CoxPH model was constructed with selected variables (i.e., clinical factors, molecular factors, and patient subtype) based on their significant and independent prognostic impact. The survival prediction performance was evaluated based on the area under the curve (AUC) with 1,000 bootstraps at a sampling rate of 0.8 on the TCGA-BRCA cohort.

### Association of CMS With Molecular Features

Differentially expressed genes (DEGs) between patient CMS were estimated (edgeR package in R, Version 3.30.3) based on the count data of the TCGA-BRCA cohort, where genes with |log_2_FC| >0.585 (FC: fold change; FC = 1.5) and FDR <0.05 were selected and visualized *via* volcano plot (EnhancedVolcano package in R, Version 1.6.0). Gene ontology (GO) and Kyoto Encyclopedia of Genes and Genomes (KEGG) pathway enrichment analysis were performed (14) (clusterProfiler package in R, Version 3.16.1) to exam the biological functions of DEGs. The total mutation number of each TCGA-BRCA sample was calculated (maftool package in R, Version 2.4.05) (15) based on MuSe (16) preprocessed mutation data. The infiltration scores of immune cells and overall immune infiltration score were estimated *via* R package “ConsensusTME” (version: 0.0.1.9000) (17), and the total T cell infiltration score was calculated according to the method introduced by Senbabaoglu et al. (18).

### Statistical Analysis

Survival differences between subtypes or groups were examined using the log-rank test. Differences in the immune cell infiltration and genomic heterogeneity (tumor mutation burden) between subtypes were analyzed using the Mann–Whitney non-parametric test. p-Value (FDR corrected if applicable) less than 0.05 was considered to be statistically significant. All analysis was performed with R (Version 4.0.2).

## Results

### Study Design and Characteristics of Patient Cohorts

**Figure 1:**
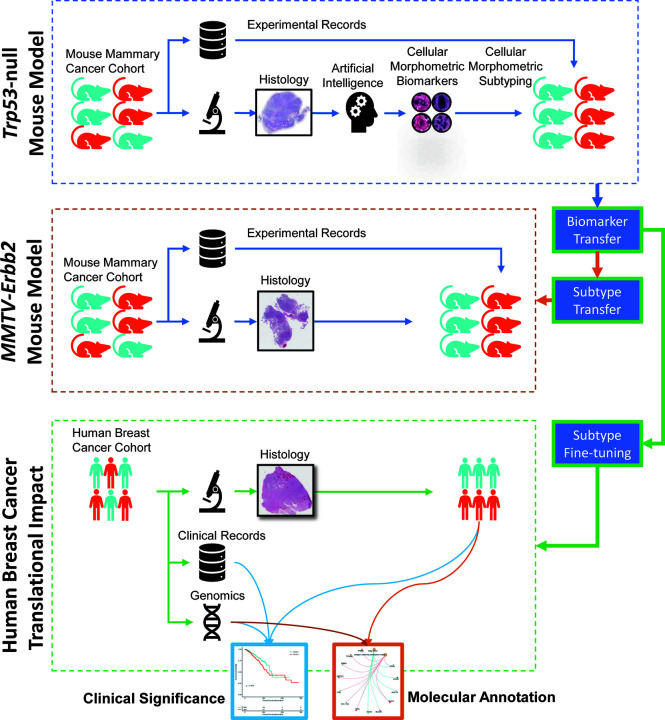
Graphical illustration of our study design with knowledge mining from one mouse mammary model and translation to another mouse mammary tumor model and human breast cancer.

The overall study design is illustrated in **Figure 1**. Specifically, we identified CMB from the whole slide images of carcinomas arising from *Trp53*-null mammary epithelium orthotopically transplanted in syngeneic wildtype mice (referred to herein as *Trp53*-null). This training cohort consisted of 154 specimens. Additional information, namely, histological tumor subtype and survival (the time from tumor detection to euthanasia, details see *Methods* section) were generated from our previous study (10) (**Supplementary Table 1**). The validation mouse mammary tumor cohort consisted of 53 specimens of mammary tumors arose in *MMTV-Erbb2* transgenic mice, referred to herein as *Erbb2* ^11^ (**Supplementary Table 2**). Additional information was the presence or absence of metastases. Human BC consisted of 1,085 diagnostic slides from 1,017 primary BC from the TCGA-BRCA cohort (**Supplementary Tables 3**, **4**).

### Identification of CMB from Trp53-Null Mouse Mammary Tumors

The AI pipeline recognized and delineated over 75 million cellular objects in the *Trp53*-null tumor training set of digital micrographs of H&E stained FFPE sections, where each cellular object was represented with 15 morphometric properties as described in our previous work (9). Next, we optimized and trained our stacked predictive sparse decomposition (SPSD) (12) model to discover CMB based on pre-quantified cellular objects randomly selected from the training mouse mammary tumor histopathological slides. After training, the pre-built SPSD model reconstructed each cellular object as a sparse activation of the pre-identified 256 CMB (**Supplementary Figure 1**), which led to the novel representation of every single cellular object as the 256-dimentional sparse code (i.e., reconstruction coefficient); and thereafter, the corresponding cellular morphometric context representation of each tumor sample in aggregation of all delineated cellular objects belonging to the same specimen.

### Correlation Between Trp53-Null Survival and CMB

**Figure 2:**
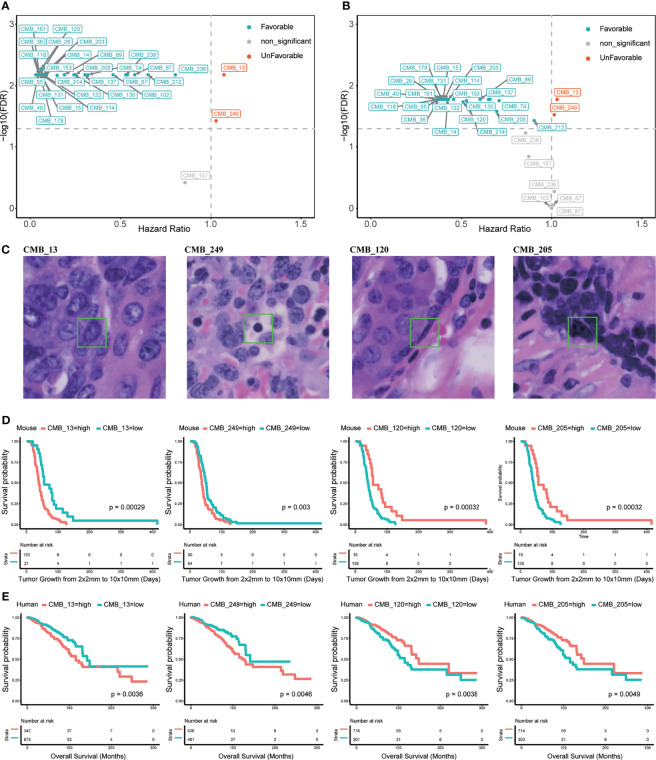
Cellular morphometric biomarkers (CMB) learned from mouse mammary tumors and extracted from human breast tumors. **(A)** Top 30 CMB learned from the *Trp53*-null cohort with most prominent variations; **(B)** Top 30 CMB extracted from the TCGA-BRCA with most prominent variations; **(C)** Examples of representative CMB learned from the *Trp53*-null cohort. **(D, E)** KM curves for representative CMB learned from the *Trp53*-null cohort, and the TCGA-BRCA cohort, respectively.

We next evaluated the association of the 256 CMB in the *Trp53*-null training set concerning survival using CoxPH analysis. This analysis revealed that 248 out of 256 CMB had a significant prognostic impact (FDR <0.05). Specifically, within the top 30 CMB with the most prominent variations, 27 were significantly and positively, and 2 were significantly and negatively associated with survival (**Figure 2A** and **Supplementary Table 5**). Representative examples of CMB demonstrated the capability of our AI pipeline in detecting interpretable cellular subtypes. Specifically, CMB_13 represents a pleomorphic tumor nucleus; CMB_249 represents a lymphocyte; CMB_120 represents the nucleus of a myoepithelial or myofibroblast; and CMB_205 represents a condensed tumor cell nucleus (**Figure 2C**).

The training mouse mammary tumor cohort was divided into two groups based on relative abundance of CMB_13, CMB_120, CMB_249, or CMB_205. Kaplan–Meier curves further confirmed significant association of these four CMB with survival (p <0.01, **Figure 2D**).

### Identification of CMS in Trp53-Null Mammary Tumors

**Figure 3:**
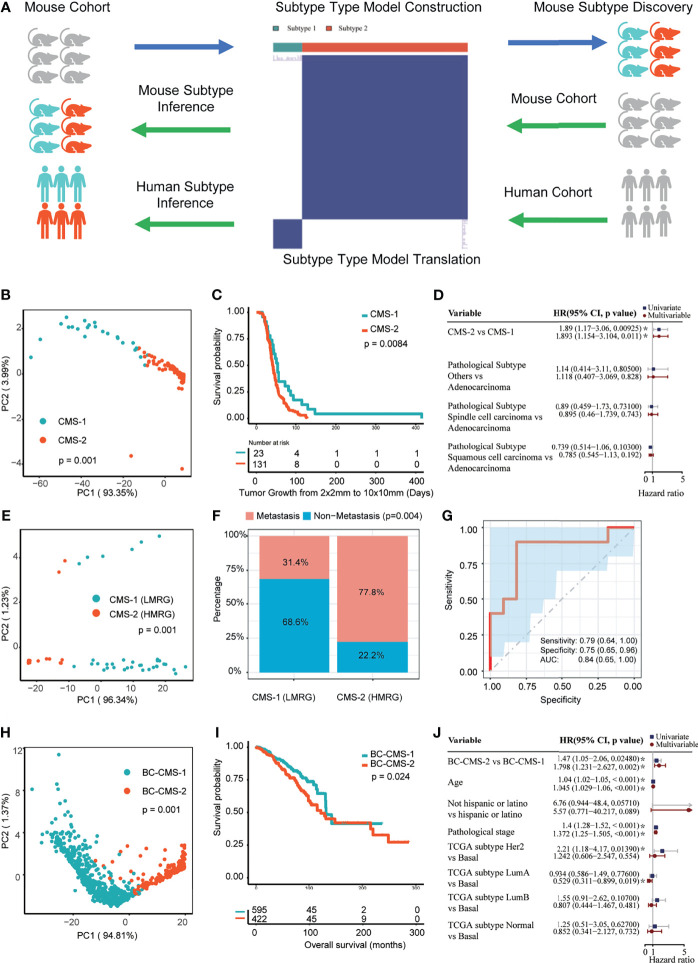
Cellular morphometric subtypes (CMS) identified from one mouse mammary tumor model are informative in another mouse mammary tumor model and provides translational impact on human breast cancer. **(A)** Consensus clustering model for CMS identification and translation from one mouse mammary tumor model to another mouse mammary tumor model and human breast cancer. **(B)** CMS-specific samples in the *Trp53*-null cohort form distinct clusters in sample-level cellular morphometric context space. **(C)** CMS-specific samples in the *Trp53*-null cohort show significant difference in survival. **(D)** CMS in the *Trp53*-null cohort is a significant and independent prognosis factor. **(E)** CMS-specific samples in the *MMTV-Erbb2* transgenic mouse mammary tumors cohort form distinct clusters in sample-level cellular morphometric context space. **(F)** CMS in *MMTV-Erbb2* transgenic mouse mammary tumors cohort is significantly enriched with metastasis presence. **(G)** Cellular morphometric biomarkers predict metastasis presence in *MMTV-Erbb2* transgenic mouse mammary tumors cohort with accuracy. **(H)** BC-CMS-specific patients in the TCGA-BRCA cohort form distinct clusters in patient-level cellular morphometric context space. **(I)** BC-CMS-specific patients in the TCGA-BRCA cohort show significant difference in OS. **(J)** BC-CMS in the TCGA-BRCA cohort is a significant and independent prognostic factor.

We next determined if the 256 CMB could stratify the training mouse mammary tumor cohort by performing consensus cluster analysis to define CMS (**Supplementary Figure 2**). Two significantly different (p = 0.0001) subtypes, CMS-1 and -2, were identified (**Figures 3A, B**). CMS-1 was characterized by higher relative abundance of 23 CMB and CMS-2 was characterized by higher relative abundance of 131 CMB. We next determined whether subtype was associated with survival using Kaplan–Meier analysis. CMS-2 tumors were significantly associated with shorter survival than subtype 1 tumors (p = 0.0084, **Figure 3C**). Importantly, the CMS adjusted for pathological subtypes (i.e., adenocarcinoma, spindle cell carcinoma, and squamous cell carcinoma) were significantly associated with survival (HR: 1.893, 95%CI: 1.154–3.104, p = 0.011; **Figure 3D**).

### Translation of CMB and CMS to Erbb2 Model

We next applied the CMB and CMS learned from the training *Trp53*-null tumor cohort to classify *Erbb2* tumors (**Supplementary Table 2**). The CMS model stratified the *Erbb2* validation mouse cohort into two CMS consisting of 35 and 18 tumors each. Moreover, the specimens of CMS-1 and 2 were clearly separated by the tumor-level cellular morphometric context representation (p = 0.001, **Figure 3E**).

CMS subtype was significantly associated with tumor metastasis in the *Erbb2* cohort (p = 0.004, **Figure 3F**). Cross-validation with logistic regression model built upon CMB positively associated with metastasis (i.e., CMB_13, OR: 1.282, 95%CI: 1.142–1.492, FDR = 0.001; and CMB_249, OR: 1.078, 95%CI: 1.022–1.143, FDR = 0.008), using 60% training samples, 40% testing samples and 100 bootstrapping iterations, predicted metastasis status with accuracy (**Figure 3G**; AUC: 0.84, 95%CI: 0.65–1.00; Sensitivity: 0.79, 95%CI: 0.64–1.00; and Specificity: 0.75, 95%CI: 0.65–0.96). In addition, representative CMB examples showed significant and consistent differences in relative abundance between metastatic and non-metastatic tumors (**Supplementary Figure 3**).

### Translation of CMB and CMS to Human Breast Cancer

We next asked if the pre-built SPSD model and CMB learned from mouse mammary tumors was applicable to human breast cancer, where the 256-dimentinal cellular morphometric context representation of each BC in the TCGA-BRCA cohort was an aggregation of all delineated cellular objects belonging to the same specimen.

Univariate Cox proportional hazard regression (CoxPH) analysis on the TCGA-BRCA cohort showed that 241 of the 256 CMB had significant prognostic association (FDR <0.05). Specifically, of the top 30 CMB with the most prominent variations, 22 were prognostically favorable (HR <1) and 2 were prognostically unfavorable (HR >1) (**Figure 2B** and **Supplementary Table 6**). It should be noted that all 22 favorable CMB were also favorable in the mouse training cohort and the 2 unfavorable CMB were also unfavorable in the mouse training cohort. Moreover, the Kaplan–Meier analysis showed significant association with the levels of each representative CMB with OS (p <0.05, **Figure 2E**). These were informative in both non-triple-negative BC (non-TNBC) patients (p <0.05, **Supplementary Figure 4A**), and triple negative breast cancer (TNBC) patients (p <0.05, **Supplementary Figure 4B**).

Based on the CMB and CMS from mouse model, we classified the TCGA-BRCA cohort into two subtypes (subtype 1: 648 patients; subtype 2: 369 patients; **Supplementary Table 3**). The patient stratification with mouse CMS model showed clear separation (**Supplementary Figure 5A**), and was further refined by re-applying consensus clustering analysis on the TCGA-BRCA cohort (**Supplementary Figure 5B**). Compared with the mouse CMS model, the refined CMS model (referred to herein as BC-CMS) led to statistically consistent patient stratification (Chi-square test, p = 9.07e−179; subtype 1: 595 patients; subtype 2: 422 patients) with slightly improved patient clusters (**Supplementary Figure 5B**). Moreover, the patient-level cellular morphometric context representation in the TCGA-BRCA cohort formed significantly separable groups in all BC patients (p = 0.001, **Figure 3H**), non-TNBC patients (p= 0.001, **Supplementary Figure 4C**), and TNBC patients (p = 0.001, **Supplementary Figure 4C**), respectively. Given the statistical consistency between mouse CMS and BC-CMS models, we used the BC-CMS on the TCGA-BRCA cohort in the rest of the study.

### Molecular Annotation Underlying BC-CMS of Human Breast Cancers

**Figure 4:**
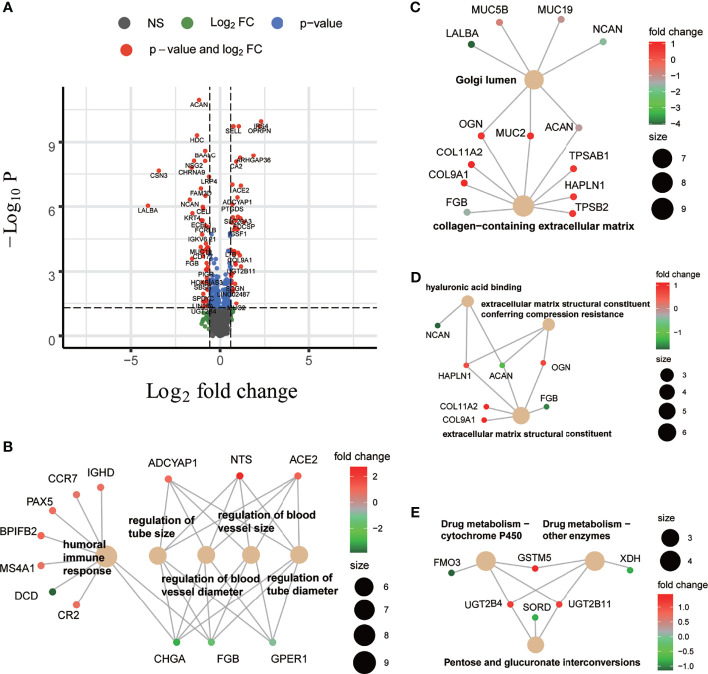
Differentially expressed genes (DEGs) between two BC-CMS and associated functional enrichment analyses. **(A)** Volcano plot depicting the differentially expressed genes with FC >1.5 and FDR <0.05. **(B)** Biological process (BP) enrichment analysis on DEGs. **(C)** Cellular component enrichment analysis on DEGs. **(D)** Molecular function enrichment analysis on DEGs. **(E)** Kyoto Encyclopedia of Genes and Genomes pathway enrichment analysis on DEGs.

To gain insight into molecular differences underlying the BC-CMS, we used available transcriptome data from the TCGA-BRCA cohort and identified a total of 111 genes that are differentially expressed between BC-CMS (|log_2_FC|>0.585, FDR <0.05, **Figure 4A** and **Supplementary Table 7**), where 50 and 61 genes were upregulated and downregulated, respectively, in subtype 2 compared to subtype 1. Gene ontology (GO) functional enrichment analysis of the differentially expressed genes (DEGs) demonstrated significant enrichment (FDR <0.05) for biological processes involved humoral immune response, regulation of tube size, regulation of blood vessel size, regulation of blood vessel diameter, and regulation of tube diameter (**Figure 4B** and **Supplementary Table 8**). Cellular component GO terms significantly enriched (FDR <0.05) in the DEGs included Golgi lumen, and collagen-containing extracellular matrix (**Figure 4C** and **Supplementary Table 9**). Molecular function GO terms (FDR <0.05) included extracellular matrix structural constituent conferring compression resistance, extracellular matrix structural constituent, and hyaluronic acid binding (**Figure 4D** and **Supplementary Table 10**). KEGG analysis indicated that DEGs were significantly enriched (FDR <0.05) in pentose and glucuronate interconversions, drug metabolism—cytochrome P450, and drug metabolism—other enzymes (**Figure 4E** and **Supplementary Table 11**). Together these findings indicate potential molecular mechanisms that differentiate the two BC-CMS including differences in tumor vascularization and immune infiltration.

### Association of BC-CMS With Tumor Immune Microenvironment

**Figure 5:**
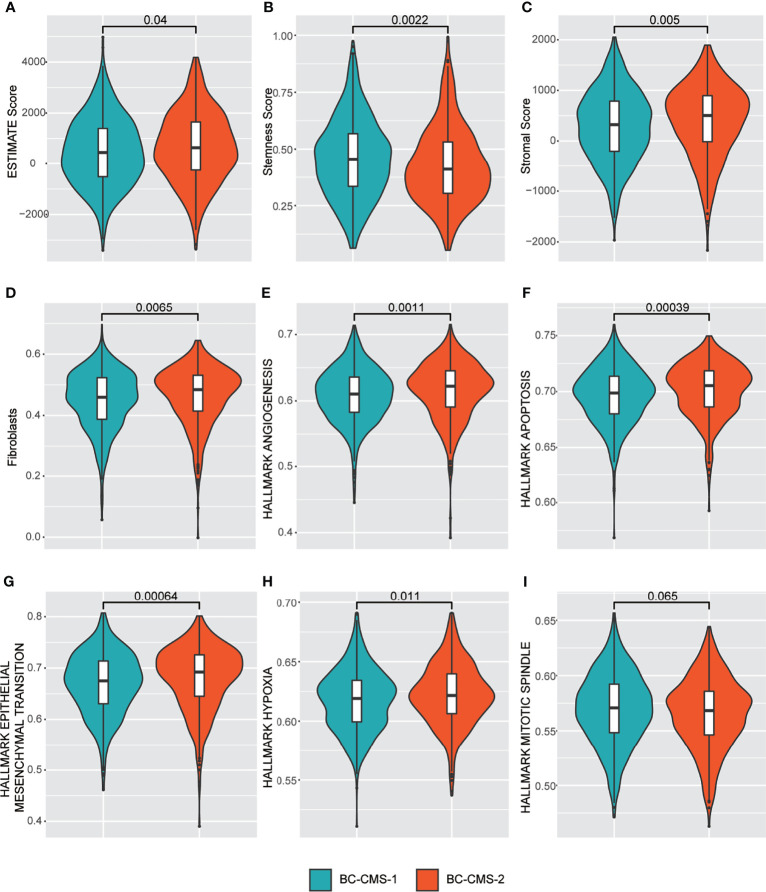
TCGA-BRCA BC-CMS shows significant differences in the relative abundance of **(A)** Estimate score (that infers tumor purity); **(B)** Stemness score; **(C)** Stromal score; **(D)** Fibroblasts; **(E)** Angiogenesis; **(F)** Apoptosis; **(G)** Epithelial mesenchymal transition; **(H)** Hypoxia; and **(I)** Mitotic spindle.

Using TCGA-BRCA expression profile data, we found that BC-CMS 2 was associated with significantly increased tumor purity (**Figure 5A**; p = 0.04), stromal score (**Figure 5C**; p = 0.005), fibroblasts (**Figure 5D**; p = 0.0065), angiogenesis (**Figure 5E**; p = 0.001), apoptosis (**Figure 5F**; p = 0.00039), epithelial–mesenchymal transition (**Figure 5G**; p = 0.00064), and hypoxia (**Figure 5H**; p = 0.011); and significantly decreased stemness score (**Figure 5B**; p = 0.0022); and marginally descreased mitotic spindle (**Figure 5I**; p = 0.065).

We also investigated the association of BC-CMS with the inferred immune microenvironment. Compared to TNBC subtype 1 patient group, TNBC subtype 2 patient group (**Supplementary Figure 6**) were characterized by inference of significantly more B cells (p = 0.032), CD4^+^ T cells (p = 0.03), CD8^+^ T cells (p = 0.042), T regulatory cells (p = 0.038), macrophages M2 (p = 0.04), endothelial cells (p = 0.045), monocytes (p = 0.019), plasma cells (p = 0.046), and immune score (p = 0.041). In contrast, the non-TNBC subtype 2 patient group, compared to the subtype 1 group (**Supplementary Figure 6**), had significantly more B cells (p = 0.048), endothelial (p = 0.017), and fibroblasts (p = 0.0042).

### Clinical Significance of BC-CMS in Human Breast Cancer

We examined the association between BC-CMS and clinical and tumor characteristics in the TCGA-BRCA cohort. Surprisingly, there was no significant association between BC-CMS and clinical or molecular prognostic factors, namely, histological type, pathological stage, PR/ER/Her2 status (**Supplementary Table 4**), indicating that CMS adds independent information.

**Figure 6:**
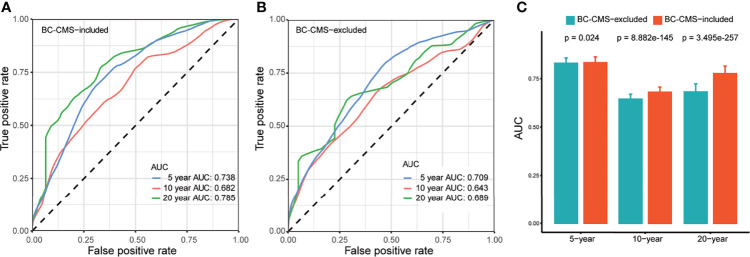
BC-CMS significantly improves prognosis prediction of BRCA patients. **(A)** ROC curves for the prediction of 5-, 10-, and 20-year overall survival of BRCA patients using all significant prognostic factors; **(B)** ROC curves for the prediction of 5-, 10-, and 20-year overall survival of BRCA patients using all significant prognostic factors except BC-CMS; **(C)** Comparison of predictive power between BC-CMS included and BC-CMS excluded models using bootstrapping strategy with 80% sampling rate and 1,000 iterations.

Kaplan–Meier analysis showed significantly shorter overall survival (OS) of subtype 2 patients compared to subtype 1 patients in all BC patients (p = 0.024, **Figure 3I**), non-TBNC patients (p = 0.018, **Supplementary Figure 4D**), and TNBC patients (p = 0.036, **Supplementary Figure 4D**). Furthermore, univariate and multivariate CoxPH models indicated the independent prognostic impact of BC-CMS in the TCGA-BRCA cohort after adjustment for other significant clinical and molecular factors, namely, age, pathological stage, and PAM50 subtype (1, 2) (HR: 1.798, 95%CI: 1.231–2.627, p = 0.002; **Figure 3J** and **Supplementary Table 12**). The combination of BC-CMS and clinical and molecular factors provided significantly improved (p <0.05, **Figure 6**) prediction of 5-, 10-, and 20-year OS compared to classical models with only clinical and molecular factors. Thus, BC-CMS adds additional prognostic value.

## Discussion

This study is aimed to develop and validate an AI-powered knowledge mining and transfer framework across mouse mammary tumor models and species (i.e., human breast cancer), which help maximize the translational impact of discoveries from mouse model to human patients. We used an AI pipeline to identify CMB from digital micrographs of diagnostic H&E histology slides of *Trp53*-null mouse mammary tumors that were used to define two CMS. These CMB and CMS were validated in the MMTV-ErbB2 transgenic mouse model. CMS-2 associated with shorter survival in *Trp53*-null tumors and development of metastasis in *Erbb2* tumors. We then translated this to digital H&E micrographs of human breast cancers from the TCGA-BRCA cohort. BC-CMS has independent prognostic significance after adjusting for other clinical and molecular factors. The application of CMS to digital images of routine workflow H&E preparations thus provides unbiased biological stratification to inform patient care.

The conservation of the cellular morphometric environment across species further validates the use of the mouse as a model to study human BC development. Mouse mammary tumor development in the *Trp53 null* model has many similarities to human breast cancer, including the progression from pre-neoplastic lesions to ductal carcinoma *in situ* to tumors of diverse histopathology and a subset of the tumors expressing the estrogen receptor (ER+) (19, 20). Herschkowitz et al. reported (8) that the transcriptional profiles of *Trp53 null* tumors could be classified into multiple molecular subtypes, namely, two basal-like, a luminal, a claudin-low, and a subtype unique to this model, which we also found (21). Notably, translation of transcriptomic signatures obtained from *Trp53* null mammary tumor model informs the diversity of human BC (21, 22). Conserved gene expression features exist between murine mammary carcinoma models and BC (7). Our study shows that CMB and CMS identified from mouse mammary tumors in the *Trp53*-null model also classify human BC and have clinical significance, supporting similarities of the biological processes in *Trp53*-null mammary tumors and human BC.

The insights gained from CMS-ML are further extended by the pragmatic considerations. Diagnostic pathology is widely practiced using FFPE preparations and standard H&E staining that are readily available throughout the world. Digital micrographs of these preparations can be shared and analyzed using our AI pipeline to provide accessible, low-cost added value in a range of clinical care settings. Indeed, a comparable approach in colorectal cancer was used to predict clinically relevant RNA expression classifiers from H&E images, providing feasible, cost-efficient biological stratification within routine workflows (23). Similarly, our data associating BC-CMS with specific molecular features suggest that further refinement of the BC-CMS may be a low-cost alternative to classify intrinsic molecular subtypes that are currently uses as clinical decision tools.

CMB identified tumor infiltrating lymphocytes, among other distinctive cell types (**Figure 2C**), that can be prognostic in BC (24). The tumor immune microenvironment is gaining traction as valuable prognostic and predictive information. Clinical trials have shown that patients with metastatic triple-negative breast cancer are more likely to respond to checkpoint inhibitors if cells express PD-L1, but the predictive value of PD-L1 expression is quite modest (25). The tumor immune microenvironment can range from hot, in which T cells are present but incapacitated or exhausted, to cold, which by definition lacks T-cell infiltration, exhibits little immune cytotoxicity, and predicts adverse outcomes in cancer patients (26). Inflamed, ‘hot’ cancers are thought to be prime targets for checkpoint immunotherapy, while so-called ‘cold’ tumors devoid of TIL are inherently resistant (27). As suggested by the association of inferred immune cell types from the TCGA transcriptomics, we anticipate further refinement of CMB could inform this aspect of cancer therapy.

In conclusion, we developed a pathology-image-based biomarker and subtype detection and translation framework that stratifies (1) an independent mouse mammary tumor cohort into groups with different metastasis risk; and (2) the TCGA-BRCA cohort into two groups with different OS associated with specific molecular features and immune infiltrate.

## Data Availability Statement

The original contributions presented in the study are included in the article/**Supplementary Material**. Further inquiries can be directed to the corresponding authors.

## Ethics Statement

The animal study was reviewed and approved by the Animal Welfare and Research Committee, Lawrence Berkeley National Laboratory, or by the Institutional Animal Care and Bioethical Committee of Universidad de Salamanca.

## Author Contributions

J-HM, MB-H, and HC conceived and designed the overall study. J-HM and HC acquired funding and co-wrote the manuscript. HC, XY, XP-L, and J-HM developed the algorithm and performed the computational analysis and statistical interpretation. RC-C, NG-S, MM-E, and JPL prepared the *MMTV-Erbb2* mouse mammary tumor cohort, and interpreted the results. JM, AS, LM, and WC prepared *Trp53-null* mammary tumor cohort, and interpreted the results. K-YJ provided pathological interpretation. All authors contributed to the article and approved the submitted version.

## Funding

This work was supported by the Department of Defense (DoD)BCRP: BC190820 (J-HM); and the National Cancer Institute (NCI) at the National Institutes of Health (NIH): R01CA184476 (HC). Lawrence Berkeley National Laboratory (LBNL) is a multi-program national laboratory operated by the University of California for the DOE under contract DE AC02-05CH11231.

## Conflict of Interest

The authors declare that the research was conducted in the absence of any commercial or financial relationships that could be construed as a potential conflict of interest.

## Publisher’s Note

All claims expressed in this article are solely those of the authors and do not necessarily represent those of their affiliated organizations, or those of the publisher, the editors and the reviewers. Any product that may be evaluated in this article, or claim that may be made by its manufacturer, is not guaranteed or endorsed by the publisher.
