# Supplementary material for: From Mouse to Human: Cellular Morphometric Subtype Learned From Mouse Mammary Tumors Provides Prognostic Value in Human Breast Cancer
Source: Front Oncol. 2022 Feb 11;11:819565. doi: 10.3389/fonc.2021.819565 (PMC8886672; doi:10.3389/fonc.2021.819565)
Supplement: Supplementary file 1 [file Image_1.pdf]

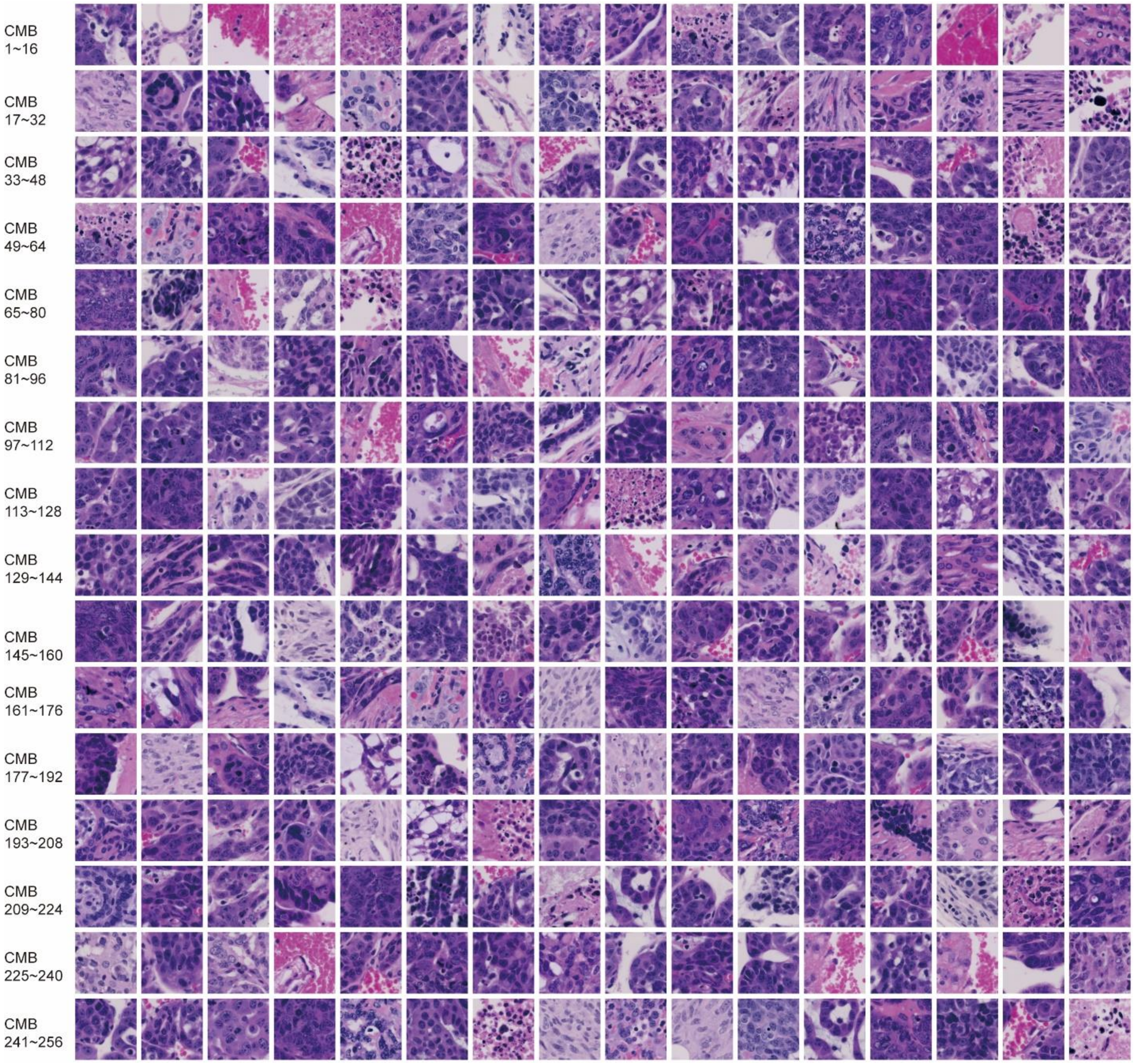

Supplementary Figure 1. Representative examples of 256 CMB learned from *Trp53*-null mouse mammary tumors.

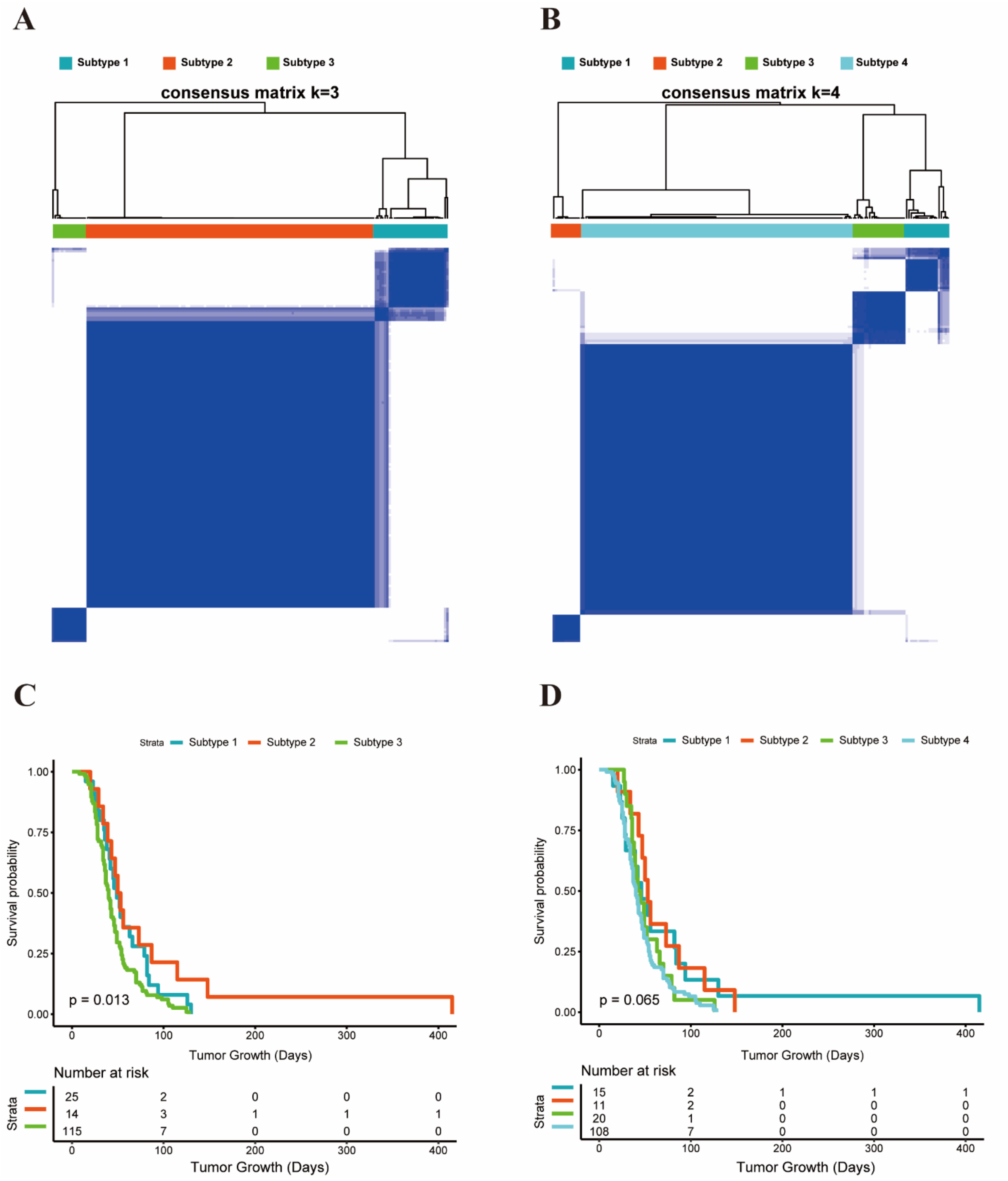

Supplementary Figure 2. Consensus clustering on the *Trp53*-null mouse mammary tumors with different number of clusters (K) and the corresponding Kaplan–Meier curves for tumor growth. **A-B**. Consensus matrix with 3 and 4 clusters, respectively; **C-D** Kaplan–Meier curves for 3 and 4 subtypes, respectively.

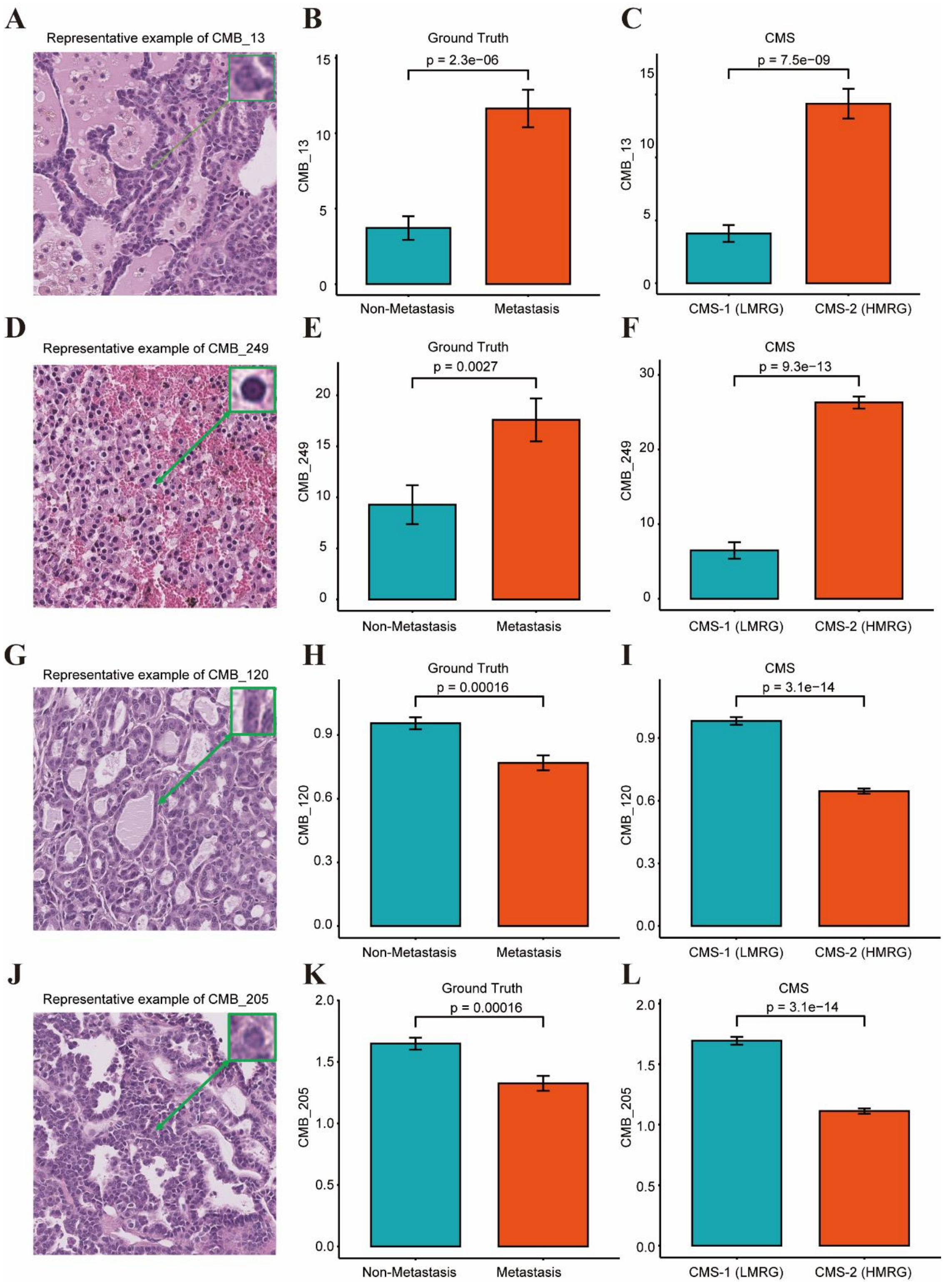

Supplementary Figure 3. Representative example of CMB_13 (**A**), CMB_249 (**D**), CMB_120 (**G**), and CMB_105 (**J**), and their significant and consistent difference in relative abundance between metastasis ground truth (**B**, **E**, **H**, **and K**) and low/high metastasis risk groups (i.e., LMRG and HMRG defined by CMS-1 and CMS-2, respectively) (**C**, **F**, **I**, and **L**).

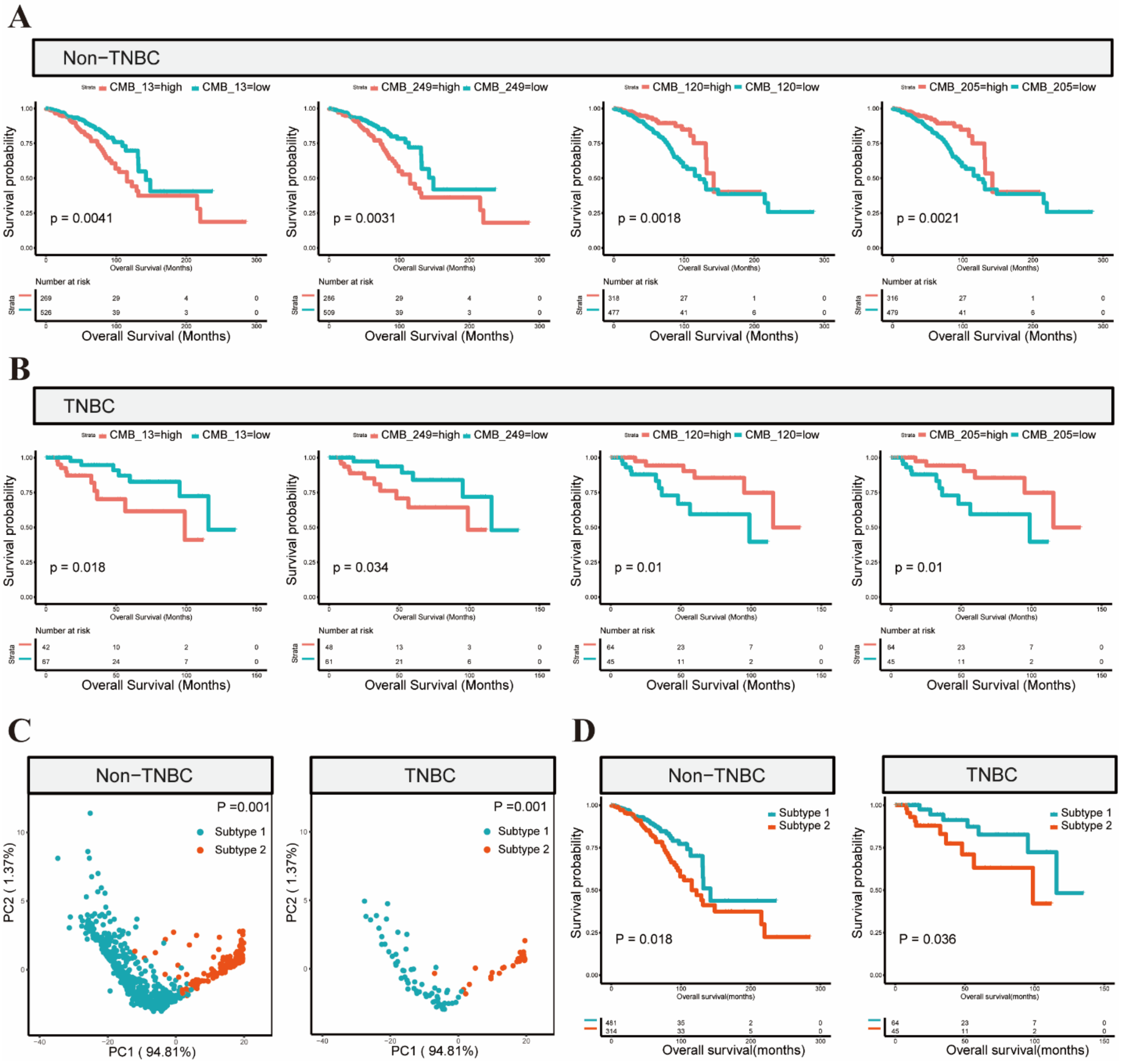

Supplementary Figure 4. BRCA patient subtypes in triple-negative (TNBC) and non-triple-negative (Non-TNBC) groups. **A-B**. KM curves for representative CMBs show consistent and significant impact on OS in Non-TNBC and TNBC groups, respectively; **C**. Subtype-specific patients in TCGA-BRCA cohort form distinct clusters in patient-level cellular morphometric context space in Non-TNBC and TNBC groups, respectively; **D**. Subtype-specific patients in TCGA-BRCA cohort show significant difference in survival in Non-TNBC and TNBC groups, respectively.

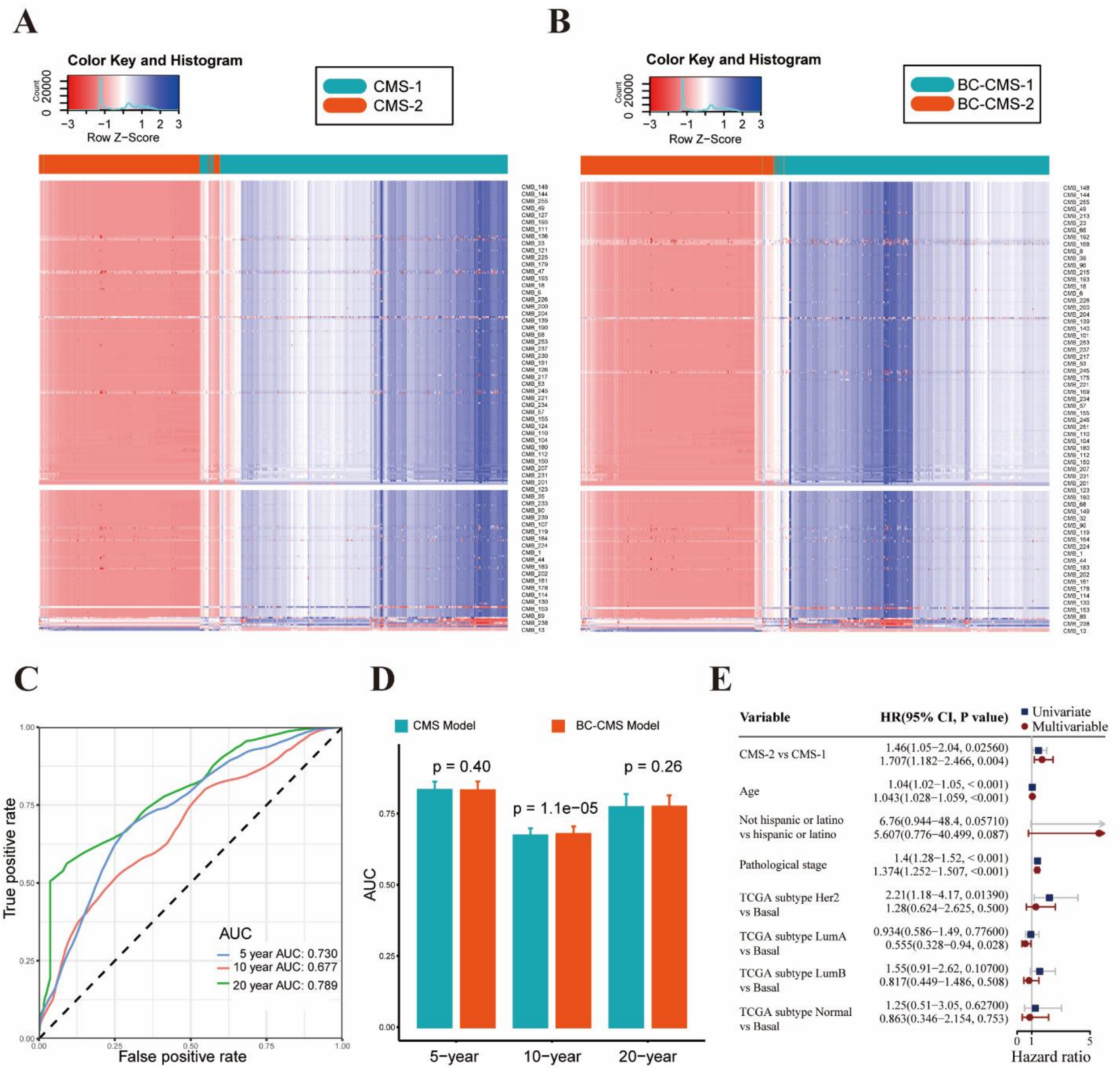

Supplementary Figure 5. **A**. BRCA patient heatmap with mouse CMS model on the TCGA-BRCA cohort; **B**. BRCA patient heatmap with BC-CMS model on the TCGA-BRCA cohort. **C**. ROC curves for the prediction of 5-,10-, and 20-year overall survival of BRCA patients using all significant prognostic factors as listed in **E**; **D**. Comparison of predictive power between BC-CMS model and mouse CMS model using bootstrapping strategy with 80% sampling rate and 1000 iterations; **E**. Similar to patient subtype from BC-CMS model as shown in **Figure 3F**, patient subtype directly predicted from the mouse CMS model is also a significant and independent prognostic factor in the TCGA-BRCA cohort.

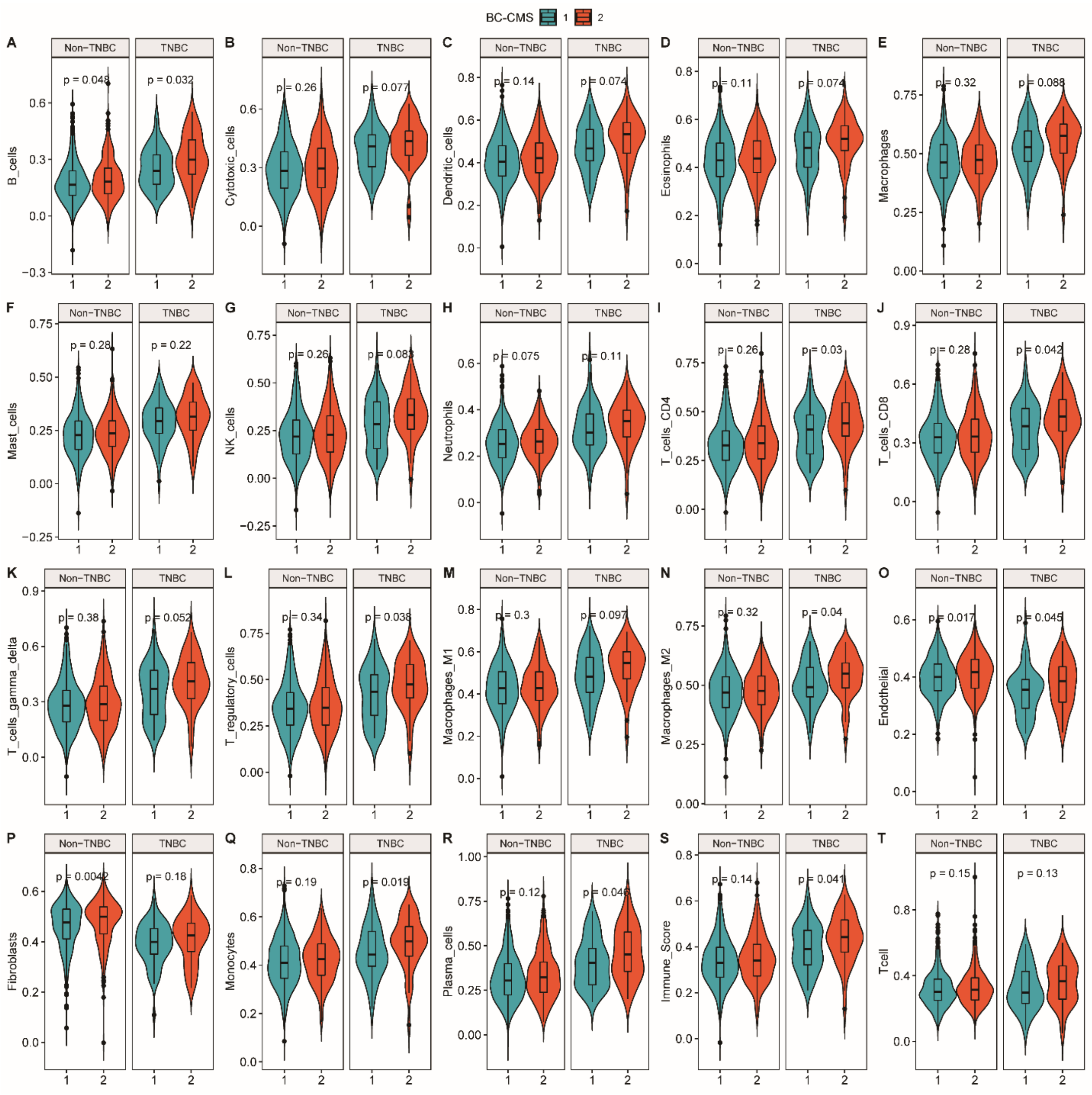

Supplementary Figure 6. BC-CMS in triple-negative (TNBC) and non-triple-negative (Non-TNBC) groups in the TCGA-BRCA cohort show significant difference in tumor microenvironments.
