# Supplementary material for: From Mouse to Human: Cellular Morphometric Subtype Learned From Mouse Mammary Tumors Provides Prognostic Value in Human Breast Cancer
Source: Front Oncol. 2022 Feb 11;11:819565. doi: 10.3389/fonc.2021.819565 (PMC8886672; doi:10.3389/fonc.2021.819565)
Supplement: Supplementary file 5 [file Table_4.docx]

**Supplementary Table 4. Clinical characteristics of patients in TCGA-BRCA cohort**

|  | **Subtype 1 (N=595)** | **Subtype 2 (N=422)** | **P value** | **Overall (N=1017)** |
| --- | --- | --- | --- | --- |
| **OS time (years)** |  |  |  |  |
| Mean (SD) | 1150 (1040) | 1440 (1380) | <0.001 | 1270 (1200) |
| Median [Min, Max] | 759 [1.00, 7110] | 1000 [1.00, 8610] |  | 867 [1.00, 8610] |
| **OS** |  |  |  |  |
| Yes | 60 (10.1%) | 82 (19.4%) | <0.001 | 142 (14.0%) |
| No | 535 (89.9%) | 340 (80.6%) |  | 875 (86.0%) |
| **Age (years)** |  |  |  |  |
| Mean (SD) | 58.7 (13.4) | 57.3 (12.4) | 0.122 | 58.1 (13.0) |
| Median [Min, Max] | 59.0 [27.0, 89.0] | 58.0 [26.0, 90.0] |  | 58.0 [26.0, 90.0] |
| **Gender** |  |  |  |  |
| FEMALE | 587 (98.7%) | 418 (99.1%) | 0.778 | 1005 (98.8%) |
| MALE | 8 (1.3%) | 4 (0.9%) |  | 12 (1.2%) |
| **Histological type** |  |  |  |  |
| Infiltrating Carcinoma NOS | 1 (0.2%) | 0 (0%) | 0.681 | 1 (0.1%) |
| Infiltrating Ductal Carcinoma | 422 (70.9%) | 305 (72.3%) |  | 727 (71.5%) |
| Infiltrating Lobular Carcinoma | 117 (19.7%) | 72 (17.1%) |  | 189 (18.6%) |
| Medullary Carcinoma | 4 (0.7%) | 1 (0.2%) |  | 5 (0.5%) |
| Metaplastic Carcinoma | 5 (0.8%) | 3 (0.7%) |  | 8 (0.8%) |
| Mixed Histology (please specify) | 15 (2.5%) | 13 (3.1%) |  | 28 (2.8%) |
| Mucinous Carcinoma | 11 (1.8%) | 6 (1.4%) |  | 17 (1.7%) |
| Other, specify | 20 (3.4%) | 21 (5.0%) |  | 41 (4.0%) |
| Missing | 0 (0%) | 1 (0.2%) |  | 1 (0.1%) |
| **Race list** |  |  |  |  |
| AMERICAN INDIAN OR ALASKA NATIVE | 1 (0.2%) | 0 (0%) | 0.02 | 1 (0.1%) |
| ASIAN | 41 (6.9%) | 16 (3.8%) |  | 57 (5.6%) |
| BLACK OR AFRICAN AMERICAN | 99 (16.6%) | 63 (14.9%) |  | 162 (15.9%) |
| WHITE | 386 (64.9%) | 332 (78.7%) |  | 718 (70.6%) |
| Missing | 68 (11.4%) | 11 (2.6%) |  | 79 (7.8%) |
| **Ethnicity** |  |  |  |  |
| HISPANIC OR LATINO | 20 (3.4%) | 18 (4.3%) | 0.788 | 38 (3.7%) |
| NOT HISPANIC OR LATINO | 465 (78.2%) | 362 (85.8%) |  | 827 (81.3%) |
| Missing | 110 (18.5%) | 42 (10.0%) |  | 152 (14.9%) |
| **PR status** |  |  |  |  |
| Indeterminate | 4 (0.7%) | 0 (0%) | 0.238 | 4 (0.4%) |
| Negative | 192 (32.3%) | 126 (29.9%) |  | 318 (31.3%) |
| Positive | 382 (64.2%) | 265 (62.8%) |  | 647 (63.6%) |
| Missing | 17 (2.9%) | 31 (7.3%) |  | 48 (4.7%) |
| **ER status** |  |  |  |  |
| Negative | 130 (21.8%) | 94 (22.3%) | 0.187 | 224 (22.0%) |
| Positive | 449 (75.5%) | 295 (69.9%) |  | 744 (73.2%) |
| Indeterminate | 0 (0%) | 2 (0.5%) |  | 2 (0.2%) |
| Missing | 16 (2.7%) | 31 (7.3%) |  | 47 (4.6%) |
| **Her2** |  |  |  |  |
| Equivocal | 110 (18.5%) | 62 (14.7%) | 0.688 | 172 (16.9%) |
| Indeterminate | 7 (1.2%) | 3 (0.7%) |  | 10 (1.0%) |
| Negative | 316 (53.1%) | 207 (49.1%) |  | 523 (51.4%) |
| Positive | 96 (16.1%) | 53 (12.6%) |  | 149 (14.7%) |
| Missing | 66 (11.1%) | 97 (23.0%) |  | 163 (16.0%) |
| **Pathological stage** |  |  |  |  |
| Stage I | 41 (6.9%) | 44 (10.4%) | 0.133 | 85 (8.4%) |
| Stage IA | 42 (7.1%) | 38 (9.0%) |  | 80 (7.9%) |
| Stage IB | 4 (0.7%) | 3 (0.7%) |  | 7 (0.7%) |
| Stage II | 2 (0.3%) | 4 (0.9%) |  | 6 (0.6%) |
| Stage IIA | 194 (32.6%) | 139 (32.9%) |  | 333 (32.7%) |
| Stage IIB | 159 (26.7%) | 83 (19.7%) |  | 242 (23.8%) |
| Stage III | 1 (0.2%) | 1 (0.2%) |  | 2 (0.2%) |
| Stage IIIA | 89 (15.0%) | 54 (12.8%) |  | 143 (14.1%) |
| Stage IIIB | 11 (1.8%) | 11 (2.6%) |  | 22 (2.2%) |
| Stage IIIC | 30 (5.0%) | 27 (6.4%) |  | 57 (5.6%) |
| Stage IV | 11 (1.8%) | 7 (1.7%) |  | 18 (1.8%) |
| Stage X | 4 (0.7%) | 7 (1.7%) |  | 11 (1.1%) |
| Missing | 7 (1.2%) | 4 (0.9%) |  | 11 (1.1%) |
| **TNBC** |  |  |  |  |
| No | 481 (80.8%) | 314 (74.4%) | 0.8 | 795 (78.2%) |
| Yes | 64 (10.8%) | 45 (10.7%) |  | 109 (10.7%) |
| Missing | 50 (8.4%) | 63 (14.9%) |  | 113 (11.1%) |
| Mean (SD) | 0.608 (0.0407) | 0.615 (0.0430) | 0.001 | 0.611 (0.0418) |
| Median [Min, Max] | 0.610 [0.446, 0.714] | 0.622 [0.392, 0.715] |  | 0.615 [0.392, 0.715] |
| Missing | 14 (2.4%) | 12 (2.8%) |  | 26 (2.6%) |
| Missing | 204 (34.3%) | 115 (27.3%) |  | 319 (31.4%) |
